# Associations between gestational weight gain and rate of infancy weight gain in Hawai‘i and Puerto Rico WIC participants

**DOI:** 10.1186/s40608-018-0219-z

**Published:** 2018-12-03

**Authors:** Cheryl L. K. Gibby, Cristina Palacios, Maribel Campos, Eunjung Lim, Jinan Banna

**Affiliations:** 1 Department of Human Nutrition, Food and Animal Sciences, College of Tropical Agriculture and Human Resources, University of Hawai‘i at Mānoa. Agricultural Sciences 216, 1955 East-West Rd, Honolulu, HI 96822 USA; 2 Department of Dietetics and Nutrition, Robert Stempel College of Public Health & Social Work, Florida International University, 11200 SW 8th Street, AHC 5–313, Miami, FL 33199 USA; 3 Dental and Craniofacial Genomics Unit, Endowed Health Services Research Center, Medical Sciences Campus, University of Puerto Rico, PO Box 365067, San Juan, PR 00936-5067 USA; 4 Department of Complementary and Integrative Medicine, Biostatistics Core Facility, John A. Burns School of Medicine, University of Hawai‘i at Mānoa. Medical Education Building 411, 651 Ilalo Street, Honolulu, HI 96813 USA

**Keywords:** Gestational weight gain, Rapid infancy weight gain, WIC

## Abstract

**Background:**

Excessive gestational weight gain and rapid infancy weight gain (RIWG) are associated with increased susceptibility to childhood obesity. Since low-income and minority children are particularly at risk, investigation of the associations between gestational weight gain and rate of infancy weight gain may inform childhood obesity prevention. This study investigated the associations between gestational weight gain and rate of infancy weight gain during the first four to six months postpartum in participants from the Special Supplemental Nutrition Program for Women, Infants, and Children (WIC) in Hawai‘i and Puerto Rico.

**Methods:**

This was a cross-sectional secondary data analysis from a text message-based intervention in WIC participants in Hawai‘i and Puerto Rico. The analysis included 80 mother/infant pairs from the control group who completed the follow-up visit when infants were four to six months old. Maternal weight, height, and gestational weight gain were self-reported. Infant weight was measured at baseline and follow-up. A proportional odds model was used to investigate the association between gestational weight gain and infancy weight gain rate (rapid or extremely rapid, on-track, or slow), adjusting for maternal age, pregravid body mass index (BMI) status, parity, and being up-to-date with infant vaccinations.

**Results:**

In comparison to recommended gestational weight gain, excessive and inadequate (under the recommended amount) gestational weight gain was associated with 77% decreased (adjusted odds ratio [AOR] = 0.23; 95% confidence interval [CI] = 0.08, 0.70; *p* = 0.01) and 71% decreased (AOR = 0.29; 95% CI = 0.09, 0.94; *p* = 0.04) odds of RIWG versus on-track or slow infant weight gain, respectively. In comparison to women with one child, women with two children (AOR = 0.31; 95% CI = 0.11, 0.87; *p* = 0.03) or three or four children (AOR = 0.24; 95% CI = 0.07, 0.88; *p* = 0.03) had significantly lower odds of RIWG versus on-track or slow infancy weight gain.

**Conclusions:**

Women with excessive or inadequate gestational weight gain had lower proportional odds of RIWG and were more likely to have slower infant weight gain than women who gained the recommended amount of weight.

**Trial registration:**

ClinicalTrials.gov Identifier; NCT02903186; September 16, 2016.

## Background

Childhood overweight and obesity is a global public health concern and has increased from 32 to 41 million children from 1990 to 2016 [1]. In the US, according to the 2011–2012 National Health and Nutrition Examination Survey (NHANES), 7.1% of infants and toddlers under the age of two years were at or above the 97.7th percentile, signifying high weight for length [2]. The 2011–2014 NHANES further indicated that 8.9% of US children aged two to five years and 17% of youth aged two through 19 years were obese [3]. Low-income and minority children are particularly at risk for obesity [4]. In 2014, 40% of US one-year-olds and 30% of two to five-year-olds participating in the Special Supplemental Nutrition Program for Women, Infants, and Children (WIC), a federally funded program for low-income women and children with gross household income at or below 185% of the US Poverty Income Guidelines [5], were overweight or obese [6, 7]. Among minority groups, Hispanic and Black children have increased odds of rapid infancy weight gain (RIWG) [8], and Native Hawaiian or Other Pacific Islander (NHOPI) children have higher weight status between two and four months of age compared to other races [9].

Although infants display the greatest variation in rates of weight gain during the first and second years after birth [10], the first six months are a critical time of rapid growth during which metabolic programming can occur, leading to increased susceptibility to obesity later in life [11–13]. Rapid weight gain during the first two years is associated with higher body mass index (BMI), greater percentage of body fat, and more total fat mass [10], as well as other problems later in life, such as overweight, obesity, high blood pressure, and diabetes [14–17]. Children who displayed catch-up growth, a compensatory mechanism for intrauterine growth restraint [10, 18] or low birth weight [19], during the first two years were heavier, taller, and had more central fat than other children [10]. Similarly, children displaying RIWG (defined as > + 0.67 change in weight-for-age z-scores) in the first two years had about 7 kg more total body fat and 3 kg greater abdominal adipose tissue at age 30–61 years (mean age 46.5 years) [20]. In Hawai‘i, it was shown that a greater change in weight between two and 24 months was associated with higher BMI at age five [9] and that rapid growth from 12 to 23 months was associated with obesity at four to five years in low-income Filipino and NHOPI children [21].

Similarly, maternal weight gain during pregnancy may have significant effects on weight of offspring [22]. Genetic, dietary, or other behavioral factors that increase gestational weight gain may program offspring weight gain by altering the fetal intrauterine environment [22]. Excessive gestational weight gain is associated with increased neonatal fat mass and body fat percentage, possibly indicating that neonatal adiposity is influenced by maternal fat accumulation and the intrauterine environment during early and mid-pregnancy [23]. One study reported mothers with greater weight gain during pregnancy had children with greater adiposity at age three as measured by BMI and skinfold thickness [22]. Other studies have concluded that excessive gestational weight gain is associated with abnormal weight in offspring, with higher risk of high BMI at ages two and eight [24], obesity at age eight [25], and increased risk of obesity in adolescence and early adulthood [26].

Therefore, excessive maternal gestational weight gain and RIWG are two important factors associated with childhood overweight and obesity. Preventing excessive gestational weight gain, a modifiable maternal risk factor, may allow for prevention of RIWG. Examination of this association may be informative for obesity prevention strategies. A positive association or trend between excessive gestational weight gain and RIWG has been reported in other studies [27–31]; however, no studies have been conducted in Hawai‘i or Puerto Rico. To our knowledge, this is the first study to assess the association in low-income women in these locations. We hypothesized that excessive gestational weight gain was positively associated with RIWG in the first four to six months postpartum in this population.

## Methods

### Participants

We conducted a cross-sectional secondary data analysis using data collected from a four-month text message-based intervention aimed at preventing excessive weight gain in low-income infants at Hawai‘i and Puerto Rico WIC clinics [32]. Eligibility criteria for mothers/caregivers included the following: at least 18 years old, able to read, owned a mobile phone with unrestricted texting capabilities, were responsible for caring for the infant, were willing to be randomized and to complete the entire study, and were willing to sign, for themselves and their infant, the written informed consent form which included a description of the study and information such as procedures, voluntary withdrawal, risks, benefits, compensation, and confidentiality. Participants were presented with the informed consent form prior to data collection. Additionally, inclusion criteria required the infant to have been born after 37 weeks of gestation, to be on a normal diet, and to be free from disabilities that hinder movement. Since the main study investigated infant feeding practices, at baseline, infants were required to have been no more than two months old to ensure that they were no more than six months old at the end of the intervention when complementary foods are recommended to be introduced in the diet. Also, infants must have had birthweight at or between the 10th and 90th percentiles as indicated by the World Health Organization (WHO) growth charts [33], thereby excluding infants who were small for gestational age who could experience compensatory growth. A convenience sample of infants (*n* = 202) and their mother/caregiver were recruited from four WIC clinics in Hawai‘i and two WIC clinics in Puerto Rico from January to April, 2016. Participants were assigned to control or intervention groups by block randomization. Detailed methods used in the trial have been published elsewhere [32].

### Measures

As described previously [34], the baseline assessment included questionnaires and infant anthropometric measurements. Data collected from the questionnaires included the following: socio-demographic information such as race, mother’s age, and infant’s gender; pregnancy- and health-related information such as maternal pregravid weight and height, weeks of gestation at delivery, weight gained during pregnancy, and infant vaccinations; food frequency information; and infant feeding practices information such as breastfeeding initiation and duration, reasons for discontinuing breastfeeding, and complementary foods used. Questionnaires and anthropometric measurements were repeated at the follow-up visit four months after the completion of the baseline assessment.

Analyses for the current study included the control group only (*n* = 100). At baseline, gestational weight gain (exposure) was assessed by self-report on the general demographics questionnaire using item, “How much weight did you gain during pregnancy?” Pregravid BMI was calculated using self-reported weight and height. Pregravid BMI was defined based on the National Institutes of Health classifications for BMI (kg/m^2^): underweight (<18.5), normal (18.5–24.9), overweight (25–29.9), obese (≥30) [35]. Based on these BMI ranges, excessive gestational weight gain was defined as weight gain above the 2009 Institute of Medicine (IOM) gestational weight gain guidelines: underweight women are recommended to gain 12.7–18.2 kg; normal weight women are recommended to gain 11.4–15.9 kg; overweight women are recommended to gain 6.8–11.3 kg; obese women are recommended to gain 5–9.1 kg [36]. Inadequate gestational weight gain was defined as weight gain under the recommended amount.

Infant weight and length were measured in duplicate by trained researchers and WIC employees at baseline and follow-up. The Hawai‘i team used Doran infant pan scales (model DS4100) and Easy Glide Bearing Infantometers (Perspective Enterprises, PE-RILB-BRG2), which were provided by the WIC clinics. The Puerto Rico team used Detecto mechanical pediatric scales (models 450, 451, 459, 459HC) and Seca Infantometer 417. Weight was measured with light or no clothes, no shoes, and a clean diaper. Infant age at follow-up ranged from four to six months. As the preferred system for analysis of anthropometric data [37], z-scores were used for assessment. Z-scores are independent of sex and age, and different age and sex groups may be combined for growth evaluation [38]. Infancy weight gain (main outcome of interest) z-scores are defined as the change in weight-for-age z-scores from baseline to follow-up, calculated using the WHO AnthroPlus online calculator [39], which is based upon WHO reference values [40, 41]. Rapid infancy weight gain is defined as a change in weight gain z-score of + 0.67 to + 1.28, representing upwards crossing of at least one percentile line on WHO standard growth charts; extremely rapid infancy weight gain is > + 1.28, representing upwards crossing of two or more percentile lines on WHO standard growth charts [10, 42–45]. Similarly, change in weight-for-age z-score between − 0.67 and 0.67 is defined as on-track infancy weight gain in which there is no crossing of percentile lines, and slow infancy weight gain occurs when weight gain z-score < − 0.67, representing downward crossing of one or more percentile lines [42].

**Fig. 1:**
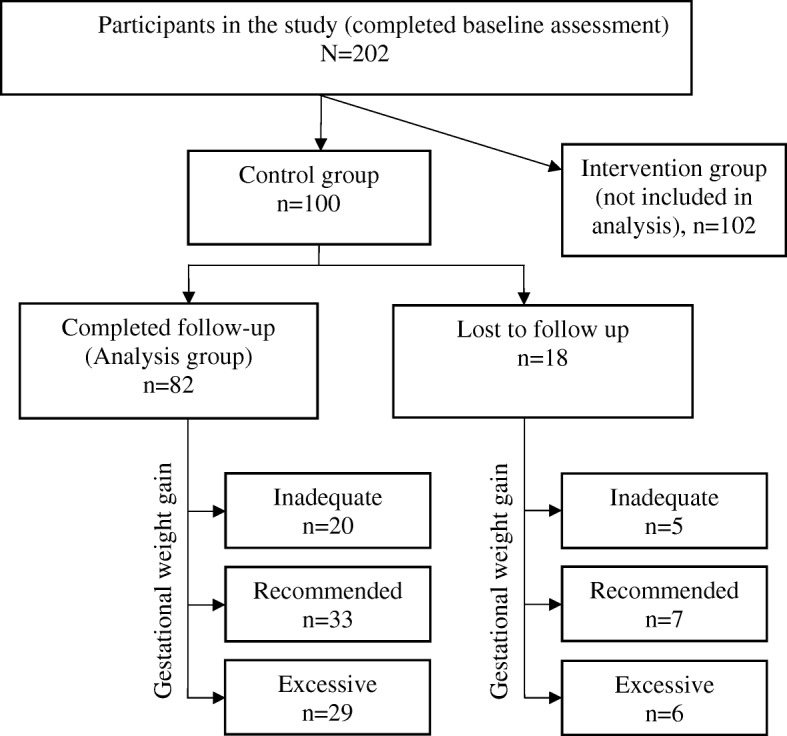
Final analysis group and participants lost to follow-up

Figure 1 describes inclusion in the analysis group. Eighty-two participants from the control group who completed the follow-up visit and had complete data were included in the final analysis group. Eighteen participants were lost to follow up: Hawai‘i (*n* = 13) and Puerto Rico (*n* = 5).

### Variables

#### Dependent variable

Rate of infancy weight gain, determined by the change in weight-for-age z-score, during the first four to six months postpartum was defined as a ternary outcome in mothers from the control group (*n* = 100) who completed the study (*n* = 82). Rapid (*n* = 12) and extremely rapid (*n* = 7) infancy weight gain were combined into one category, simply referred to as rapid infancy weight gain [43, 44], due to the small sample size. In addition, the distributions between rapid and extremely rapid infancy weight gain on the following covariates were comparable to each other.

#### Independent variable

Maternal gestational weight gain was categorized as “recommended,” “inadequate,” or “excessive,” based on the IOM gestational weight gain guidelines [36]. Excessive gestational weight gain was defined as weight gain above the IOM gestational weight gain guidelines, and inadequate gestational weight gain was defined as weight gain under the recommended amount.

#### Possible covariates

Variables tested as possible covariates included site (Hawai‘i or Puerto Rico), mother’s race/ethnicity, mother’s age (by years: 18–24, 25–31, 32–39), mother’s education level (less than college, some college, college degree of higher), parity (1, 2, 3 or 4), use of prenatal vitamins during pregnancy, pregnancy complications (such as diabetes, hypertension, or anemia), use of vitamins while breastfeeding, weeks of gestation at delivery, pregravid BMI status (underweight, normal, overweight, obese), infant gender, and being up-to-date with infant vaccinations. Based upon the US Office of Management and Budget standards [46], race/ethnicity was categorized into the following groups: Hispanic, NHOPI, American Indian or Alaska Native, Asian, Black or African American, and White. Most of these covariates were selected based upon their inclusion in previous studies that investigated similar associations [31, 47]. However, in regards to the vaccination covariate, it was reported that, in rats, the pertussis vaccine increased glucose-induced insulin secretion [48]. Therefore, since infants routinely receive the pertussis vaccine during the first six months after birth [49], being up-to-date with infant vaccinations was included as a possible covariate.

#### Statistical analyses

For baseline characteristics, descriptive statistics were presented using frequencies and percentages, or using means and standard deviations. Chi-squares tests, Fisher’s exact tests, and analysis of variance (ANOVA) were conducted to investigate the bivariate associations or comparison between baseline characteristics and rate of infancy weight gain. Fisher’s exact test was used to examine the bivariate association between rate of infancy weight gain (rapid, on-track, or slow) and gestational weight gain (recommended, inadequate, or excessive).

To determine the association between gestational weight gain and RIWG, an adjusted proportional odds regression was conducted.

From the bivariate analyses, demographic variables with a *p* value of < 0.15 were included in the adjusted proportional odds regression [50, 51]. The covariates in the proportional odds model were pregravid BMI status (combined as underweight/normal (UN) or overweight/obese (OWOB)), being up-to-date with infant vaccinations, parity, and maternal age. For all other analyses, including a chi-square score test for the proportional odds assumption to determine goodness of fit, a *p*-value of < 0.05 was considered statistically significant. Analyses were performed using SAS 9.4 (Cary, NC).

## Results

Thirty-seven participants (45.1%) from Hawai‘i and 45 participants (54.9%) from Puerto Rico were included in the final analysis (*n* = 82). Characteristics of the control group, final analysis group, and group that was lost to follow-up are shown in Table 1. Comparing participants in the final analysis group (n = 82) with those who did not complete the study (*n* = 18), no statistically significant differences were found in subject characteristics except site, education, and use of prenatal vitamins. Women who were in Hawai‘i (*p* = 0.04), who did not take prenatal vitamins during pregnancy (*p* = 0.03), or who had less than a college education (*p* = 0.02) were more likely to have been lost to follow-up.

**Table 1:** Distribution of select maternal and infant characteristics, n (%)

	Total control (*n* = 100)	Final analysis (*n* = 82)	Lost to follow-up (*n* = 18)	*p*-value
Site				0.037
Hawai‘i	50 (50.0)	37 (45.1)	13 (72.2)	
Puerto Rico	50 (50.0)	45 (54.9)	5 (27.8)	
*Maternal factors*				
Pregravid BMI (mean [SD])	26.1 [6.9]	26.3 [7.0]	24.8 [6.6]	0.340
Underweight or normal	56 (56.0)	44 (53.7)	12 (66.7)	
Overweight or obese	44 (44.0)	38 (46.3)	6 (33.3)	
Age (mean [SD])	27.0 [5.0]	26.9 [4.7]	27.4 [6.5]	0.958
18–24 years	33 (33.0)	27 (32.9)	6 (33.3)	
25–31 years	47 (47.0)	39 (47.6)	8 (44.4)	
32–39 years	20 (20.0)	16 (19.5)	4 (22.2)	
Race^a^/ethnicity				
Hispanic	60 (60.0)	51 (62.2)	9 (56.3)	0.655
Native Hawaiian or Other Pacific Islander	17 (17.0)	12 (14.6)	5 (27.8)	0.179
Asian	20 (20.0)	14 (17.1)	6 (33.3)	0.118
American Indian or Alaska Native	4 (4.0)	3 (3.7)	1 (5.6)	0.555
Black or African American	12 (12.0)	11 (13.4)	1 (5.6)	0.689
White	45 (45.0)	40 (48.8)	5 (27.8)	0.105
Education				0.019
Less than college	42 (42.0)	31 (37.8)	11 (61.1)	
Some college	22 (22.0)	22 (26.8)	0 (0)	
College degree or higher	36 (36.0)	29 (35.4)	7 (38.9)	
Parity				0.112
1	38 (38.0)	32 (39.0)	6 (33.3)	
2	43 (43.0)	34 (41.5)	9 (50.0)	
3 or 4	19 (19.0)	16 (19.5)	3 (16.7)	
Use of prenatal vitamins	98 (98.0)	82 (100.0)	16 (88.9)	0.031^b^
Pregnancy complications	41 (41.0)	31 (37.8)	10 (55.6)	0.166
Took vitamins while breastfeeding	53 (53.0)	42 (51.2)	11 (68.8)	0.319
Gestational age (weeks; mean [SD])	38.9 [1.0]	38.9 [1.0]	38.9 [1.1]	0.844
GWG (kg; mean [SD])	12.7 [5.5]	12.6 [5.5]	13.0 [5.9]	0.759
*Infant factors*				
Male	52 (52.0)	40 (48.8)	12 (66.7)	0.169
Female	48 (48.0)	42 (51.2)	6 (33.3)	
Up-to-date with vaccinations	81 (81.0)	65 (79.3)	16 (88.9)	0.512

Note: Column percentages; p-value was obtained from comparison analysis between final analysis vs lost to follow-up. For categorical variables, chi-square tests or Fisher exact tests were conducted and two-sample t-tests were conducted for continuous variables

^a^Includes all races for mixed participants

Forty-four women (53.7%) were underweight or normal weight prior to pregnancy, and 38 women (46.3%) had overweight or obese pregravid BMI. The majority of women were Hispanic (62.2%) and among all races, White was the most prevalent (48.8%). Fifteen percent of mothers were NHOPI. All of the women used prenatal vitamins and most (79.3%) were up-to-date with infant vaccinations.

Mean gestational weight gain was 12.6 kg (SD = 5.5). Thirty-three women (40.2%) gained the recommended amount of weight during pregnancy, 20 women (24.4%) gained inadequate amounts, and 29 (35.4%) women had excessive gestational weight gain. At baseline, all except one infant’s weight-for-age z-score fell within − 2 to + 2 on the WHO growth chart, which is the range considered “normal” based upon the statistical definition of the central 95% on a normal curve. [38] During the first four to six months postpartum, 19 infants (23.2%) experienced RIWG, 42 infants (51.2%) experienced on-track weight gain, and 21 infants (25.6%) had slow weight gain.

As shown in Table 2, almost half (48.5%) of women who gained the recommended amount of weight during pregnancy had infants who displayed on-track weight gain that did not cross over percentile lines on the WHO growth charts (change in weight-for-age z-score between − 0.67 and 0.67). More than half (58.6%) of women with excessive gestational weight gain had infants who displayed on-track weight gain. Of mothers who gained excessive weight during pregnancy, more infants displayed slow infancy weight gain (31.0%) than RIWG (10.3%). The majority of infants who displayed RIWG (63.2%) came from mothers who gained the recommended amount of weight during pregnancy.

**Table 2:** Results of bivariate analysis by rate of infancy weight gain

Variable	Rate of infancy weight gain, *n*(%)			*p*-value
	Rapid 19 (23.2)	On-track 42 (51.2)	Slow 21 (25.6)	
Site				0.615
Hawai‘i	7 (18.9)	21 (56.8)	9 (24.3)	
Puerto Rico	12 (26.7)	21 (46.7)	12 (26.7)	
Gestational weight gain				0.108
Recommended	12 (36.4)	16 (48.5)	5 (15.2)	
Inadequate	4 (20.0)	9 (45.0)	7 (35.0)	
Excessive	3 (10.3)	17 (58.6)	9 (31.0)	
Pregravid BMI				0.067
Underweight	2 (28.6)	2 (28.6)	3 (42.9)	
Normal	8 (21.6)	21 (56.8)	8 (21.6)	
Overweight	6 (31.6)	12 (63.2)	1 (5.3)	
Obese	3 (15.8)	7 (36.8)	9 (47.4)	
Race^a^/ethnicity				
Hispanic	12 (23.5)	26 (51.0)	13 (25.5)	0.995
Native Hawaiian	2 (28.6)	2 (28.6)	3 (42.9)	0.441
Other Pacific Islander	3 (50.0)	3 (50.0)	0 (0)	0.161
Native Hawaiian or Other Pacific Islander	4 (33.3)	5 (41.7)	3 (25.0)	0.654
Asian	2 (14.3)	8 (57.1)	4 (28.6)	0.741
American Indian or Alaska Native	1 (33.3)	1 (33.3)	1 (33.3)	0.796
Black or African American	1 (9.1)	7 (63.6)	3 (27.3)	0.580
White	11 (27.5)	20 (50.0)	9 (22.5)	0.622
Parity				0.142
1	12 (37.5)	15 (46.9)	5 (15.6)	
2	5 (14.7)	19 (55.9)	10 (29.4)	
3 or 4	2 (12.5)	8 (50.0)	6 (37.5)	
Use of prenatal vitamins				
Yes	19 (23.2)	42 (51.2)	21 (25.6)	
Pregnancy complications				0.693
No	11 (21.6)	28 (54.9)	12 (23.5)	
Yes	8 (25.8)	14 (45.2)	9 (29.0)	
Took vitamins while breastfeeding				0.921
No	7 (20.6)	17 (50.0)	10 (29.4)	
Yes	11 (26.2)	21 (50.0)	10 (23.8)	
(No response given)	1 (16.7)	4 (66.7)	1 (16.7)	
Education				0.274
Less than college	9 (29.0)	11 (35.5)	11 (35.5)	
Some college	4 (18.2)	13 (59.1)	5 (22.7)	
College degree or higher	6 (20.7)	18 (62.1)	5 (17.2)	
Maternal age (years)				0.047
18–24	11 (40.7)	11 (40.7)	5 (18.5)	
25–31	5 (12.8)	20 (51.3)	14 (35.9)	
32–39	3 (18.8)	11 (68.8)	2 (12.5)	
Infant gender				0.257
Male	10 (25.0)	23 (57.5)	7 (17.5)	
Female	9 (21.4)	19 (45.2)	14 (33.3)	
Up-to-date with vaccinations				0.061
No	6 (35.3)	10 (58.8)	1 (5.9)	
Yes	13 (20.0)	32 (49.2)	20 (30.8)	

Note: Row percentages; *p*-value was obtained from chi-square test or Fisher’s exact test for categorical variable and analysis of variance for continuous variable

^a^Includes all races for mixed participants. Each race/ethnicity variable was defined as Yes if a participant marked the race/ethnicity on the questionnaire

Table 2 also presents the results for the bivariate analysis, which showed the association between rate of infancy weight gain and the following variables: site, race/ethnicity, pregnancy complications, took vitamins while breastfeeding, education, gestational age at birth, and infant gender (none of these associations were statistically significant). Variables with *p* < 0.15 were included in the final adjusted proportional odds cumulative logit model: pregravid BMI (*p* = 0.067), parity (*p* = 0.142), maternal age (*p* = 0.047), and up-to-date with infant vaccinations (*p* = 0.061).

Table 3 presents the results from the final proportional odds model. In the bivariate analysis, Fisher’s exact test showed the association between gestational weight gain and the rate of infancy weight gain was not statistically significant (*p* = 0.108). However, in the adjusted model, the association between gestational weight gain and RIWG was statistically significant. In comparison to recommended gestational weight gain, excessive gestational weight gain was associated with 77% decreased odds of RIWG versus on-track or slow infant weight gain (adjusted odds ratio (AOR) = 0.23; 95% CI = 0.08, 0.70; *p* = 0.01). That is, women with excessive gestational weight gain were more likely (4.3 times the odds) to have slower infant weight gain than women who gained the recommended amount of gestational weight.

**Table 3:** Proportional odds model using rate of infancy weight gain as the dependent variable

Independent variable	Estimate (SE)	*p*-value	AOR (95% CI)	
			Rapid vs on-track or slow	Slow vs rapid or on-track
Intercept				
Rapid vs on-track or slow	1.16 (0.69)	0.093		
Rapid or on-track vs slow	4.00 (0.83)	<0.001		
Gestational weight gain				
Recommended	Reference			
Excessive	-1.46 (0.57)	0.010	0.23 (0.08, 0.70)	4.32 (1.42, 13.14)
Inadequate	-1.24 (0.60)	0.039	0.29 (0.09, 0.94)	3.45 (1.07, 11.17)
Maternal age (years)				
18-24	Reference			
25-31	-0.60 (0.57)	0.289	0.55 (0.18, 1.67)	1.83 (0.60, 5.54)
32-39	0.02 (0.67)	0.975	1.02 (0.28, 3.77)	0.98 (0.27, 3.61)
Pregravid BMI				
Underweight/normal	Reference			
Overweight/obese	0.30 (0.50)	0.555	1.35 (0.50, 3.61)	0.73 (0.28, 1.99)
Parity				
1 child	Reference			
2 children	-1.16 (0.52)	0.026	0.31 (0.11, 0.87)	3.20 (1.15, 8.93)
3 or 4 children	-1.43 (0.66)	0.031	0.24 (0.07, 0.88)	4.17 (1.14, 15.28)
Up-to-date with infant vaccinations				
No	Reference			
Yes	-1.15 (0.60)	0.056	0.32 (0.10, 1.03)	3.15 (0.97, 10.21)

Estimate: Ordered log-odds regression coefficient, *SE* Standard error of the estimate, *AOR* adjusted odds ratio, *CI* confidence interval

In comparison to recommended gestational weight gain, inadequate gestational weight gain was associated with 71% decreased odds of RIWG versus on-track or slow infant weight gain (AOR = 0.29; 95% CI = 0.09, 0.94; *p* = 0.04). In other words, women with inadequate gestational weight gain were more likely (3.5 times the odds) to have slower infant weight gain than women who gained the recommended amount of gestational weight.

The adjusted model (Table 3) also indicated that, in comparison to women with one child, women with two children (AOR = 0.31; 95% CI = 0.11, 0.87; *p* = 0.026) or three or four children (AOR = 0.24; 95% CI = 0.07, 0.88; *p* = 0.031) had significantly lower odds of RIWG versus on-track or slow infancy weight gain. Although not statistically significant, women who were up-to-date with infant vaccinations had 68% decreased odds of RIWG versus on-track or slow infancy weight gain (AOR = 0.32; 95% CI = 0.10, 1.03; *p* = 0.056).

The goodness-of-fit test for the proportional odds assumption was used to validate our model; the *p*-value was 0.64, indicating good fit.

## Discussion

In contrast to our hypothesis, excessive gestational weight gain was associated with decreased, not increased, odds of RIWG. The majority of infants with RIWG (63.2%) were from mothers who achieved recommended gestational weight gain. Therefore, preventing excessive gestational weight gain may not be associated with the odds of RIWG. The latter would need to be addressed separately in prevention programs. This finding was in contrast to the results from a number of studies in diverse populations that revealed a positive association between the two [27–31]. For example, a study in China reported excessive gestational weight gain was associated with RIWG across all BMI categories [27]. The difference in results between the studies could have resulted from differences related to geography and/or ethnicity/race, such as diet, environment, or physical stature. For example, Asian adults have higher percentage of body fat than Whites of the same BMI, age, and sex [52]. In the current study, almost half of the participants were White and only 17% were Asian, whereas 100% of the population were Chinese in the aforementioned study. Other studies have reported differences in the relationship between percent body fat and BMI not only among different ethnic groups, but also among ethnicities/races in different geographic regions, such as between American Caucasians and European Caucasians and between Chinese in New York, Beijing, and Hong Kong [53, 54]. Therefore, the results of the current study may be reflective only of the study population, and more research on the factors and mechanisms behind cultural, racial, and geographical differences in the association between gestational weight gain and rate of infancy weight gain may lead to public health strategies that are more effective and better tailored to meet the needs of different populations.

In comparison to recommended gestational weight gain, both excessive gestational weight gain and inadequate gestational weight gain, separately, were associated with higher odds of slow infancy weight gain versus RIWG or on-track infancy weight gain. Another study of normal birthweight infants reported that slow infancy weight gain between ages eight weeks and nine months was followed by RIWG; however, children did not return to mean reference weights until age 13 years and they remained lighter and shorter than their peers throughout childhood [55]. Since the current study followed infant weight gain only up until six months of age, future studies should observe rates of weight gain beyond this period to see if similar patterns hold true in this population.

The current study found that 25.6% of infants experienced slow infancy weight gain. Nine infants with slow infancy weight gain (including four infants from mothers with excessive gestational weight gain and three infants from mothers with inadequate gestational weight gain) fell into the category of “failure to thrive” (FTT), defined as a child under two years whose weight crosses two major percentiles downward on a standardized growth chart [56]. Like RIWG, slow infancy weight gain may be associated with adverse health conditions and must be addressed to prevent unfavorable outcomes later in life. Slow infancy weight gain, especially when severe enough to be considered FTT, may be a sign of feeding problems, malnutrition, or of other physiological conditions that lead to inadequate calorie intake, inadequate calorie absorption, or increased calorie requirements [56]. FTT is associated with decreased developmental skills, lower height and weight, lower verbal intelligence, lower social maturity, and increased behavioral disturbances [56, 57]. Therefore, excessive or inadequate gestational weight gain may be an important risk factor for developing adverse health conditions related to FTT, and are important to address in pregnant women.

In comparison to first-time mothers with only one child, multiparous women with two to four children had approximately 3–4 times the odds of slow infancy weight gain versus RIWG or on-track infancy weight gain. First-time mothers had higher odds of rapid infancy weight gain. Likewise, a study conducted in the United Kingdom reported infants of first-time mothers displayed RIWG during the first year and, thereafter, were heavier and taller than infants from multiparous mothers [58]. A similar finding was reported in primiparous mothers in Denmark whose infants gained more weight during the first year than infants of multiparous women [59]. Different parenting behaviors between first-time mothers and multiparous mothers may influence infant nutrition [58]. A study in Australia reported that first-time mothers failed to demonstrate an understanding of the rationale supporting the WHO guidelines to exclusively breastfeed for the first six months, nor did they comprehend well the signs of readiness for solid foods [60]. Formula feeding and feeding-to-schedule may lead to overfeeding and are associated with RIWG [61]. Likewise, early introduction of solid foods along with shorter duration of breastfeeding may partially explain greater infant weight gain [59]. Therefore, women may benefit from nutrition and infant care education during their first pregnancies to prevent RIWG and during subsequent pregnancies to prevent slow infancy weight gain and FTT.

Many studies have shown that excessive gestational weight gain is associated with increased risk for obesity [24–26, 62]. This association has public health implications, as excessive gestational weight gain is not uncommon in the US. In 2015, 48% of uniparous women who gave birth to a full-term infant in the US had excessive gestational weight gain [63]. The current study found that more than one-third (35.4%) of the women in the final analysis gained excessive gestational weight, making it an important risk factor to address in this population.

Along with epidemiological evidence, the physiological mechanisms driving excessive gestational weight gain to metabolically program offspring weight gain towards overweight or obesity [62] are important to consider in obesity prevention strategies. Potential mechanisms involved in metabolic imprinting through excessive gestational weight gain include the following: 1) higher levels of maternal circulating insulin during the third trimester [64] leads to increased levels of adipocyte development and fat storage in the fetus, 2) excessive maternal adipose tissue accumulation increases low-grade chronic inflammation and circulating adipokines, possibly creating an inflammatory environment for the fetus and promoting excess body fat accumulation [65], 3) fetal metabolic programming is influenced by epigenetic changes brought on by maternal diet [66, 67]. Based upon these mechanisms, one prevention strategy may involve providing at-risk women with vigorous nutrition/diet counseling and accountability along with frequent blood glucose monitoring, if applicable.Although mainly a measure of energy balance, weight-for-age has been used most often as the metric in studies on infancy growth and later obesity [68]. However, a study that examined the differences in association between adult overweight/obesity and infant weight-for-age versus weight-for-length measurements suggested that weight-for-length during the first two years is a more comprehensive metric for infant growth and provides a stronger, more consistent association with overweight/obesity in young adulthood [69]. However, using weight-for-length versus weight-for-age measurements, the study reported similar odds ratios (OR) for being overweight at age 20–29 years based on rapid growth (≥ + 0.67 z-score) compared to non-rapid growth from birth to six months (OR 1.67 vs. 1.69) and from birth to three months (OR 1.13 vs. 1.07) [69]. Therefore, since the current study only examined infants up to six months of age, and considering the preponderance of previous studies using weight-for-age, weight-for-age was chosen as the metric. However, future studies that look at growth beyond six months may benefit from using weight-for-length measurements.

Weight gain during infancy plays an important role in overall growth and disease risk throughout life [10, 55]. Considering the results presented, weight gain in excess of or below the recommended amounts set forth by the 2009 IOM guidelines may lead to adverse health outcomes related to slow infancy weight gain and subsequent growth patterns. Preventing excessive or inadequate gestational weight gain may work simultaneously to prevent slow infancy weight gain in this population of low-income WIC participants. These findings may be helpful for health promotion and obesity prevention strategies and programs for women in WIC in Hawai‘i and Puerto Rico.

### Limitations of the study

Our study has several limitations. First, although the majority of participants in the final analysis group was Hispanic, making 77% of the final analysis group Hispanic or partly NHOPI, the sample size was small and may have caused bias in the results, such as under or overestimation of the associations. Therefore, larger studies, especially with a greater percentage of NHOPI participants, should be conducted to further investigate the associations described in this study. Second, our results are not generalizable to women beyond those participating in the study due to the inherent bias of convenience sampling. However, this study provides new information that may inform future studies. Third, although 95% of American adults in 2018 own cell phones [70] and 73% of cell phone owners use text messaging [71], according to the Pew Research Center, it is possible that the participants in the current study were at a slightly higher income level than the average WIC population since one of the eligibility criteria was that participants owned a mobile phone. However, household income information was not collected during the main study and our analysis could not include stratification within the WIC low-income bracket. Fourth, maternal height, pregravid weight, and gestational weight gain were self-reported. Fifth, although researchers were trained in proper techniques and documentation of measurements, having multiple researchers taking anthropometric measurements may have introduced human error. Additionally, 11 participants from Hawai‘i (29.7%) had infant anthropometric measurements taken in-part or in-full by WIC staff, whose training differed from the research team. All measurements in Puerto Rico were taken by the research team.

Lastly, it is possible that other factors that were not considered in the current analysis mediated the association between excessive gestational weight gain and RIWG. It was recently reported that, as mediators, breastfeeding duration and birth weight accounted for up to 76% of the total effect of gestational weight gain on weight-for-age z-scores [47]. The current study investigated the change in weight-for-age z-scores as indicative of infant weight gain, rather than single point measurements, but it is notable that these variables could have mediated the current observations. It is also possible that the associations observed in the current study may have changed had it been conducted over a longer period. In the aforementioned study, it was reported that the direct effects of gestational weight gain on weight-for-age z-scores and BMI z-scores were not significant until children were six years old [47]. Therefore, since birth weight and breastfeeding duration may be important mediators between gestational weight gain and anthropometric measures during infancy and early childhood [47], it would be prudent to include these variables in future analyses and studies.

## Conclusions

In low-income WIC participants in Hawai‘i and Puerto Rico, excessive gestational weight gain did not increase susceptibility to RIWG; however, both variables are risk factors for childhood obesity and collectively affected more than half of the women/infants in this sample. Preventing excessive and inadequate gestational weight gain may benefit infant health by preventing slow infancy weight gain and FTT. Achieving the recommended amount of gestational weight gain and keeping an infant’s weight gain on track are important to prevent malnutrition and undesirable metabolic programming. The results of this study should be interpreted within the context of the limitations noted but may provide direction for the development of strategies for obesity prevention and gestational weight gain management.

## Abbreviations

ANOVA: Analysis of variance
AOR: Adjusted odds ratio
BMI: Body mass index
CI: Confidence interval
FTT: Failure to thrive
IOM: Institute of Medicine
NHANES: National Health and Nutrition Examination Survey
NHOPI: Native Hawaiian or Other Pacific Islander
OWOB: Overweight/obese
RIWG: Rapid infancy weight gain
UN: Underweight/normal
WHO: World Health Organization
WIC: Special Supplemental Nutrition Program for Women, Infants, and Children
